# Gel Shrinkage in Discontinuous Electrophoresis: How to Stabilize the Electrolyte Boundary in Epitachophoresis—Part 1—Gel Selection

**DOI:** 10.1021/acsomega.5c08736

**Published:** 2025-11-20

**Authors:** Vanda Kocianová, Ivona Voráčová, Doo Soo Chung, František Foret

**Affiliations:** † 86852 Ústav Analytické Chemie Akademie Věd České Republiky, Veveří 97, Brno 602 00, Czech Republic; ‡ Department of Biochemistry, Faculty of Science, Masaryk University, Kamenice 5, Brno 625 00, Czech Republic; § Department of Chemistry, Seoul National University, Seoul 08826, Republic of Korea

## Abstract

Gel electrophoresis is typically performed in a single electrolyte system. During the development of epitachophoresis for large-volume DNA concentration, which employs a discontinuous electrolyte system, we found that some gels tend to shrink significantly as the boundary between the leading electrolyte (LE) and trailing electrolyte (TE) moves along the gel. Effective stabilization of this boundary is crucial for analyte focusing, particularly in systems processing tens of milliliters of a sample. This study systematically evaluated various gel stabilization media—including agarose-based gels (NEEO (no electroendosmosis), IsoGel, pulsed-field electrophoresis gel) and polyacrylamide gels—based on their ability to maintain a stable LE/TE boundary, minimize gel shrinkage, and maximize DNA recovery. Agarose gels with low electroosmotic flow were optimized by adjusting the gel concentration, electrolyte composition, and pH and by incorporating Ba^2+^ ions to reduce gel deformation caused by thermal and electrokinetic effects. Computer simulations highlighted pH gradients at the LE/TE interface as a key factor contributing to gel shrinkage. The study also revealed that careful control of the buffer composition and pH, especially when Tris, Bis-Tris, and Bis-Tris propane counterions are used, is essential for stable separation and reproducible DNA recovery. Optimal conditions for agarose gels yielded up to 100% DNA recovery, as confirmed by fluorescence-based quantification and capillary electrophoresis. Polyacrylamide gel demonstrated mechanical stability without shrinkage; however, significant sieving effects hindered the effective concentration of large DNA fragments, limiting its applicability. Overall, agarose gels designed for pulsed-field electrophoresis and optimized NEEO agarose formulations provided the best balance of stability, low analyte interaction, and high recovery efficiency for epitachophoretic DNA concentration. This work summarizes practical approaches to LE/TE interface stabilization, which is critical for large-scale biomolecular separations by epitachophoresis.

## Graphical abstract

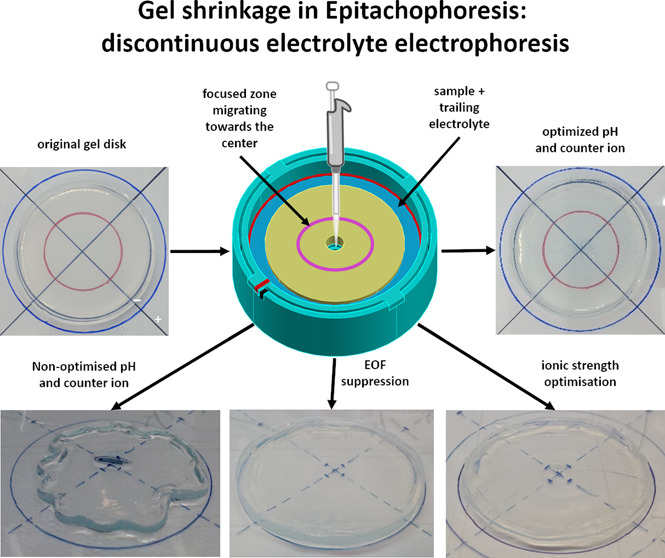

## Introduction

Epitachophoresis (ETP) is an advanced electrophoretic technique derived from isotachophoresis (ITP), distinguished by its unique circular design.^1^ One of the primary advantages of ETP is its ability to efficiently separate, concentrate, and purify large volumes of complex biological samples—ranging from hundreds of microliters to several milliliters—in approximately 1 h. The technique is also notable for its straightforward scalability, simple device setup, and flexibility in handling a wide range of charged analytes.^2–4^

A critical factor influencing the performance of both ITP and ETP is stabilization of the interface between the leading electrolyte (LE) and the trailing electrolyte (TE). This interface is essential for the formation of distinct, focused analyte zones based on the electrophoretic mobilities of the analytes.^5^ A stable LE/TE boundary ensures high resolution, prevents zone dispersion, minimizes turbulence, and enhances the reproducibility. Achieving such stabilization in capillary separations typically involves optimizing the electrolyte composition, incorporating gels or viscous media, maintaining precise control of the electric field, and designing appropriate channels or capillaries.^5^

In the context of ETP, stabilization of the LE/TE boundary presents a greater challenge due to the method’s circular design, which significantly enlarges the interface area compared to that in a traditional capillary-based ITP system. Without proper stabilization media, the LE and TE readily mix, preventing the interface formation and compromising separation and concentration efficiency.^1,2^ Various strategies have been explored to stabilize the LE/TE boundary, including the use of gels such as agarose and polyacrylamide (PAA),^1,4^ foamed polymers, microparticles,^6^ and custom 3D-printed structures.^2^ Among these, gels are particularly attractive due to their widespread use in electrophoretic and bioanalytical techniques, their ease of preparation, and their tunable physical and chemical properties.

In this study, we evaluate the performance of several gel types with differing physicochemical properties and explore modifications to the electrolyte composition to enhance the stability. The separation of model dyes and DNA recovery was used as a criterion to assess the effectiveness of each condition. Special attention was given to preventing gel shrinkage, particularly in agarose-based media, by optimizing the electrolyte composition and additives. Ultimately, we identify conditions that enable efficient DNA concentration and recovery, demonstrating the critical role of stabilization media in the success of epitachophoretic separations.

## Experimental/Materials and Methods

Chemicals used for electrolytes: l-histidine hydrochloride 1-hydrate (98,5–101,0%) and l-histidine (98,5–101,0%) were purchased from PanReac AppliChem, ITW Reagents (Castellar del Vallès, Spain; Darmstadt, Germany), *N*-tris­(hydroxymethyl)­methyl-3-aminopropanesulfonic acid (TAPS, 99.5%), and (1,3-bis­[tris­(hydroxymethyl)-methylamino]­propane) (Bis-Tris propane, BTP, 99%) were purchased from Sigma-Aldrich (St. Louis, MO, USA). Tris­(hydroxymethyl) aminomethane (Tris, 99.9%) and 2-[bis­(2-hydroxyethyl)­amino]-2-(hydroxymethyl)­propane-1,3-diol (Bis-Tris, 99%) were purchased from Carl Roth (Karlsruhe, Germany), and 35% hydrochloric acid was purchased from Lach-Ner (Brno, Czech Republic). Anionic dyes—Patent Blue V sodium salt, 1,8-dihydroxy-2-(4-sulfophenylazo)-naphthalene-3,6-disulfonic acid trisodium salt (SPADNS, ≥80%), and sucrose—were purchased from Sigma-Aldrich. SYBR Gold, used for visualizing DNA ladders, was obtained from Invitrogen (Carlsbad, CA, USA). The low-molecular-weight dsDNA ladder (10 fragments from 50 to 766 bp) and 1 kb DNA ladder were purchased from New England BioLabs (Ipswich, MA, USA). Agarose NEEO ultra quality ROTIgarose with low electroendosmosis was purchased from Carl Roth. IsoGel agarose was purchased from Lonza Group Ltd., Basel, Switzerland. Agarose for pulsed-field electrophoresis running gel was obtained from Sigma-Aldrich (USA). A ROTIPHORESE gel 30 (37.5:1 - acrylamide: bis­(acrylamide)), TEMED (≥99%, p.a.), and ammonium peroxodisulfate (APS, ≥98%, p.a.) used for the preparation of polyacrylamide gel were from Carl ROTH, Germany. Barium nitrate (Ba­(NO_3_)_2_) was purchased from VEB Laborchemie, Germany. 1% solution of hexamethylethyl cellulose was purchased from Villa Labeco, Slovakia. Sodium chloride was purchased from Penta Chemicals Unlimited, Czech Republic.

The epitachophoretic device was designed in Autodesk Autocad 3D modeling software and fabricated by injection molding (Fathom manufacturing, Oakland, USA). The stainless-steel wire ring electrode (stainless steel 1.4301, Hobby Dráty, Czech Republic) and 0.3 × 20 mm Pt wire (SAFINA, Vestec, Czech Republic) were used as electrodes. A Slide-A-Lyzer mini dialysis cup (2000 Da MWCO, Thermo Fisher Scientific, USA) was utilized for sample collection. The device for epitachophoresis, as described previously,^4^ is shown in Figure 1. Briefly, the polypropylene ETP device, with outer dimensions of 100 mm × 100 mm × 30 mm, was fabricated by injection molding (Fathom Manufacturing, Oakland, USA). The stainless-steel circular electrode was fixed on the edge of the circular separation compartment and connected via a banana plug to the power supply as a cathode. The Pt wire electrode was also fixed on the edge of the circular separation compartment and connected via a banana plug to the power supply as an anode. The collection cup was made by cutting off a mini dialysis cup with a semipermeable membrane and placing it in the central collection well.

**Figure 1:**
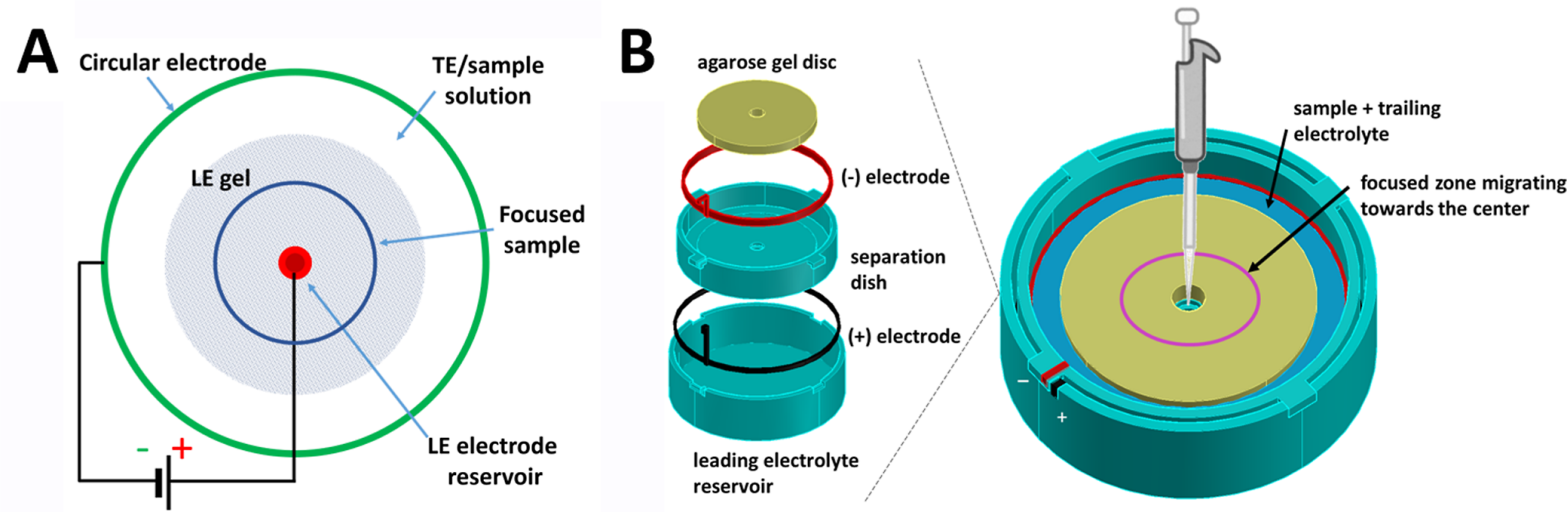
Principle of epitachophoresis (A) and scheme of the device (B).

Epitachophoretic experiments were performed in a discontinuous (two-electrolyte) buffer system comprising the LE and TE for the separation of anions, both prepared in deionized water. The actual composition of the electrolytes, as well as the preparation of the stabilizing gels, is described in conjunction with the experiments. The device was set up for the experiment according to the following procedure. The LE reservoir and collection cup were filled with LE buffer. The disk, stabilizing the LE/TE boundary, was placed in the center of the device and covered by either hydrophilic foil (GelBond Film Sheets by Lonza, USA) or PDMS foil (SYLGARD 184 Silicone Elastomer, Dow Corning, USA) to prevent evaporation. The sample solution mixed with the TE buffer was poured into the space between the gel disk and the ring electrode on the edge of the upper vessel. The power supply was connected via banana plugs and ran at a constant power of 4 W with the starting voltage of 300–349 V. The concentration process took between 30 min and more than 1 h, depending on experimental conditions. When the focused zone of the sample entered the collection cup, the power was switched off and the sample was collected by pipetting it out from the collection cup for further analysis.

Two sizes of gel disks were used. The first, with a diameter of 74 mm and a height of 4 mm, was prepared from 20 mL of LE and an agarose mixture. The second, with a diameter of 67 mm and a height of 8 mm, was prepared from 30 mL of LE and an agarose mixture. Both were poured into a homemade mold of the required size. All types of agarose gel disks were prepared by using the same procedure. An appropriate amount (according to the prepared gel concentration) of agarose powder was mixed with LE of the required concentration. The solution was boiled for 30 s, agarose was poured into a homemade plastic mold, and left to cool down at laboratory temperature. The 6% polyacrylamide (PAA) gel disks were prepared by mixing 4 mL of 30% Rotiphorese gel with 16 mL of LE, 20 μL of TEMED, and 150 μL of 10% APS. The resulting solution was poured into a homemade plastic mold and left to polymerize. Additional components, such as Ba­(NO_3_)_2_ or SYBR Gold, were added after mixing all gel components in the case of PAA gel, and, after boiling and before pouring into the molding, in the case of agarose gels. After polymerization, the gel disk was transferred to the epitachophoretic device.

Optical detection of color dyes and pictures of gels before and after focusing were taken using a mobile phone camera (Samsung Galaxy S4) under daylight or by a mobile phone (Samsung Galaxy S9 Plus) in a photobox (Foldioplus). Fluorescence images of the SYBR Gold-DNA complex were visualized using blue LED excitation and the yellow-orange photographic filter (040 Yellow/orange B + W, Schneider Optics Hauppauge, NY) placed in front of the camera objective.

The DNA recovery after epitachophoretic concentration was evaluated using a Qubit fluorometer (Invitrogen, Carlsbad, CA, USA). The composition of DNA fragments after the concentration process was controlled by a Bioanalyzer (Agilent, Waldbronn, Germany). This control was performed to monitor possible DNA fragment losses caused by the sieving effect of the gels or by leakage through the semipermeable membrane of the collection cup.

## Results and Discussion

In discontinuous electrophoresis, agarose gel shrinkage can occur due to the ionic and pH differences between the stacking and resolving buffers, leading to osmotic imbalances during electrophoresis. The higher ionic strength and lower pH of the LE, compared to the sample and TE zones, can result in contraction of the agarose gel, similar to water evaporation.^7^ Additionally, prolonged electrophoresis at high voltages can increase local heating, further promoting gel dehydration and shrinkage. Such shrinkage can distort the migration pattern of the sample zones and affect resolution during discontinuous agarose gel electrophoresis.^8^ While the effects of evaporation can be minimized by using an insulating cover (e.g., a glass plate), the ionic and osmotic gradients in discontinuous buffer systems, which can induce shrinkage resembling that caused by drying, are more difficult to counteract. It is worth mentioning that gel shrinkage has also been observed in PAA gels; however, in that case, the shrinkage was caused by the gel inhomogeneities created during the radical polymerization process.^9^ Typical examples of agarose gel shrinkage after an epitachophoretic run are shown in Figure 2.

**Figure 2:**
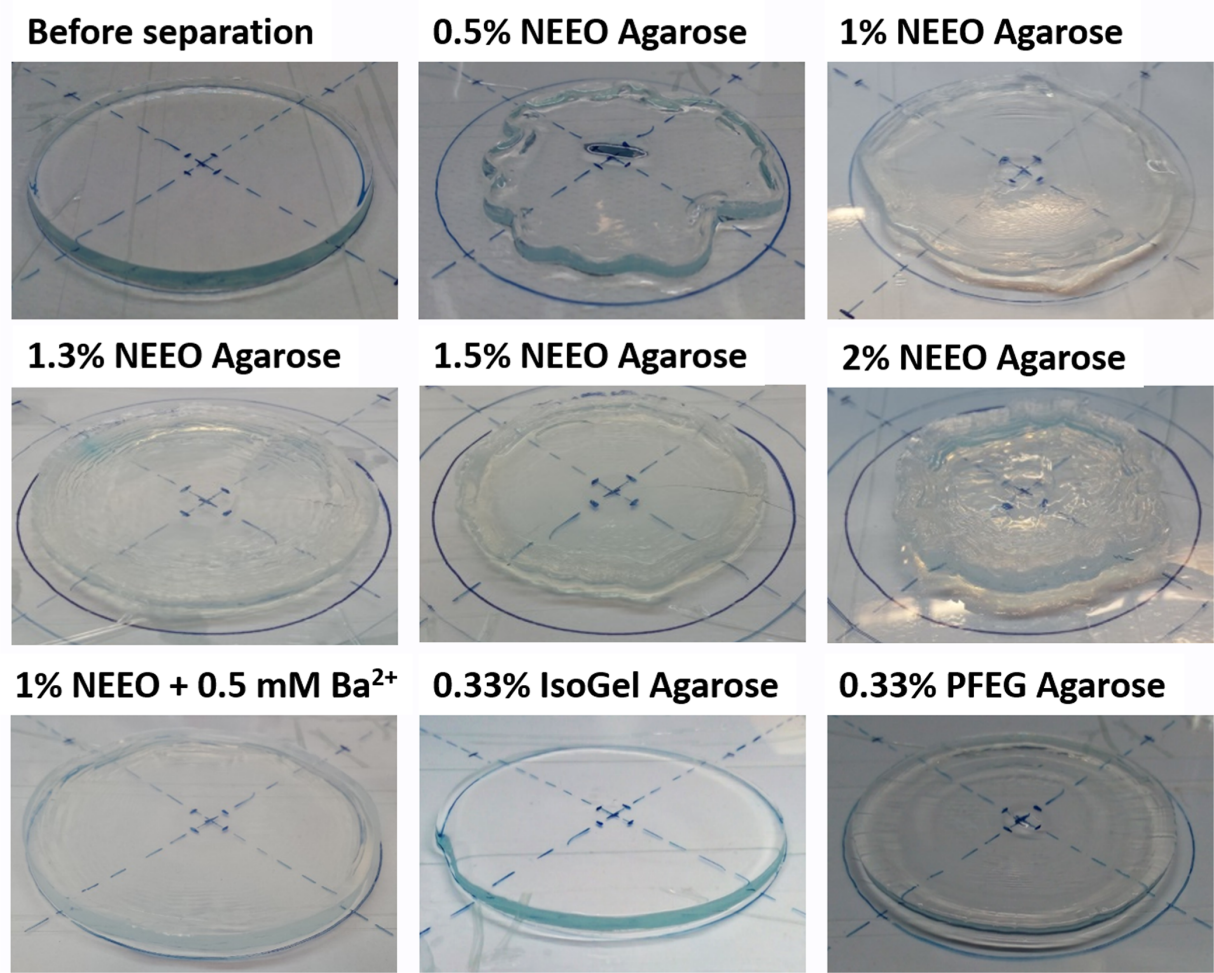
Shrinkage and deformation of various agarose gel disks after the epitachophoretic separation process, LE: 20 mM HCl-His (pH 6.25), TE: 10 mM TAPS-Tris (pH 8.3).

ETP employs a discontinuous electrolyte system composed of an LE and a TE. Unlike in capillary electrophoresis, which is suitable for the analysis of submicroliter sample volumes, ETP is designed to process samples in the tens of milliliters range.

The requirements for suitable stabilization media are multifaceted. They should have large pore sizes to avoid sieving effects that could hinder the migration of biomolecules, exhibit minimal interaction (adsorption or chemical reaction) with analytes, and maintain high chemical and mechanical stabilities throughout the separation process. Additionally, the stabilization media should be simple and quick to prepare, safe to handle, and nontoxic. Minimal analyte adsorption is especially critical, as it can cause peak broadening or even complete analyte loss. Stability during separation is also essential to ensuring reproducibility, particularly with respect to analyte collection times. Finally, the ease of preparation and use is important for practical, routine analytical workflows.

In our initial experiments, we attempted to stabilize the LE/TE boundary using a perforated plastic ring and increasing the LE buffer density via sucrose addition. However, this approach proved to be inadequate. Although the analyte zone initially formed at the ring upon the application of an electric field, it rapidly dispersed due to convection in the free solution. This method may be marginally applicable to devices designed for small samples (∼1 mL), where a porous physical barrier separates the running buffers. In such compact systems, 3D-printed stabilization barriers,^2^ semipermeable membranes, or large-pore filters can serve as effective barriers.^6^ However, this approach is not suitable for larger systems where diffusion and convection dominate mass transport. After these experiments, hydrophilic gels common in electrophoresis were selected as a potential stabilization media for the LE/TE boundary in ETP. We tested several gel types with varying physicochemical properties and evaluated their performance based on stability, sharpness of separated zones, and DNA recovery.

### Agarose-Based Gels

Initial tests focused on NEEO agarose, a low electro-osmotic flow (EOF) gel. Separation was performed using 20 mM HCl, titrated with histidine to pH 6.25 as the LE and 10 mM TAPS-Tris (pH 8.3) as the TE. The lowest functional gel concentration was found to be 0.5%, below which the gel lacked mechanical integrity and was challenging to handle. Significant gel shrinkage and deformation were observed (Figure 2), although the dye and fluorescent DNA zones remained sharp. DNA recovery using a low-molecular-weight ladder, measured by the Qubit fluorometer, exceeded 80%, and the presence and ratio of DNA fragments were confirmed using chip-based capillary gel electrophoresis with laser-induced fluorescence detection.^1^

NEEO agarose concentrations ranging from 0.5% to 2% were tested, with the lowest deformation observed at 1.3% (Figure 2). Interestingly, gel deformation had little impact on DNA recovery or fragment distribution. Unfortunately, it influences the separation. There are two main problems. One is the volume change between the gel boundary and the reservoir wall that can cause the lack of TE. The second, the gel is not shrunk in only one dimension, but in all dimensions. So, there is a risk that electrolyte flows over or under the thin, deformed gel into the collection cup and destroys the separation.

We considered several hypotheses to explain the agarose gel shrinkage. First, localized overheating at the gel center was suspected to cause thermal degradation. Temperature measurements confirmed that under 6 W (130/210 V, 46/30 mA, start/end value) of applied power, the surface temperatures at the gel center reached up to 75 °C. Reducing power to 2 W (70/167 V, 28/12 mA, start/end value) limited the temperature to below 50 °C but doubled the separation time from 60 to 120 min. A modified voltage program ultimately reduced center temperatures to below 45 °C while maintaining reasonable run times; however, temperature reduction alone did not eliminate gel shrinkage. Therefore, overheating was eliminated as a cause of the gel deformation.

Second, although NEEO agarose is classified as a low EOF matrix (EOF <0.12), residual charged groups remain on the polysaccharide chains, resulting in EOF. Further experiments were conducted using IsoGel agarose (nondetectable EOF) and agarose designed for pulsed-field gel electrophoresis (PFGE) (EOF <0.08). Both gels exhibited significantly reduced or no shrinkage after separation (Figure 2).

To further address EOF-induced shrinkage, Ba^2+^ ions were added to the LE buffer to react with negatively charged groups (such as sulfonic or thiol groups) on the agarose surface. Ba^2+^ ions were previously used for the elimination of EOF in fused-silica capillaries^10^ and partially in PAA gel,^11^ but not in combination with agarose-based gels. Various concentrations of Ba­(NO_3_)_2_ (0.2–10 mM) were tested during gel preparation and were also added to the gel and LE reservoir. The optimal condition was a 1% agarose gel with 0.5 to 2 mM Ba^2+^, which minimized gel shrinkage without extending separation time (see Table 2, Figure S2). Higher concentrations of agarose hardened the gel, causing brittleness or cracking. Lower or higher concentrations of Ba^2+^ ions were less effective in preventing shrinkage.

Interestingly, minimal gel shrinkage was also observed when biological fluids were used, especially when a physiological solution (0.9% NaCl) was added to the TE/sample mixture. Thus, the relationship among shrinkage, concentration, and NaCl content was systematically studied. Figure 3 presents a traffic light representation of gel shrinkage. Photos of the gel after ETP concentration for all measured combinations of agarose concentration and NaCl addition are shown in Figure S1. For samples containing up to 5 mL of physiological solution, NEEO agarose concentrations of 1.5 and 2% provided optimal stability. However, higher agarose concentrations limited the gel’s porosity, impeding the migration of even small molecules such as organic dyes and causing DNA fragments to separate into individual zones.

**Figure 3:**
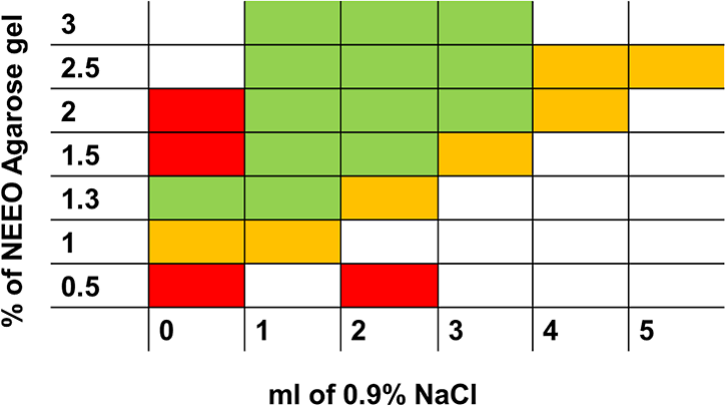
Dependence of NEEO agarose gel shrinkage and deformation during epitachophoretic separation on the gel concentration and addition of physiological solution, LE: 20 mM HCl-His (pH 6.25), TE: 10 mM TAPS-Tris (pH 8.3).

A review of the literature^8,12–15^ led us to hypothesize that the observed gel shrinkage could also be caused by conductivity and/or pH changes at the LE/TE interface. Several studies have reported gel shrinkage or deformation under applied electric fields.^8,12–16^ Most of these investigations focused on PAA gels,^12–15^ with only a few addressing agarose gels.^8,16^ In particular, structural changes in unbuffered agarose gels have been described, where ion migration from both ends of the gel under an electric field generates pH gradients that correlate with macroscopic gel shrinkage and alignment.^8^

To explore this further, we performed a series of computer simulations to examine how the composition of the LE and TE buffers influences pH and conductivity changes or even jumps at the LE/TE interface. Special attention was paid to the pH of the electrolytes and the choice of buffering cations. Several cations were evaluated as the histidine cation was found to interfere with DNA quantification by UV/vis spectrophotometry. While histidine does not affect fluorescence-based DNA detection methods, it strongly absorbs at 260 and 280 nm, making it unsuitable for absorbance-based purity assessments, which rely on the 260/280 nm ratio.

As a result, Tris, Bis-Tris, and BTP were selected as alternative buffering cations. These cations possess buffering capacities within the relevant pH range (Figure S3) and exhibit negligible absorbances at 260 and 280 nm. There are several useful papers describing the evolution of the zones in discontinuous electrophoresis.^17–19^ In this work, the calculations were conducted using the program Simul,^20^ covering various combinations of LE and TE compositions, including different cations and pH values (Table S1, Figure S4). Both pH and conductivity gradients at the LE/TE interface were monitored. Since the conductivity gradient closely mirrored the pH gradient, only the pH profiles are shown in the simulations (Figures 3 and S4) for clarity. We believe that the pH gradient at the LE/TE interface is a key factor contributing to gel shrinkage.

To validate the proposed hypothesis, 0.5% NEEO agarose gels incorporating various counterions and pH conditions were prepared. Epitachophoretic separation of dyes was performed, and gel shrinkage was systematically visually evaluated by a comparison of the original gel with the gel after the ETP process. A black circle in Figure 5 represents the original size of the gel. Electrolytes containing identical counterions were prepared at neutral pH (6.4, 7.0, 7.4, and 7.7) and basic pH (8.0, 8.3). Experiments conducted at pH 6.4 were unsuccessful in mitigating gel shrinkage and were thus restricted to LE buffer. For the TE buffer, pH values below seven were deemed unsuitable for DNA separation, the target analyte in this study. A series of experiments covering multiple pH values for each counterion was performed. Comprehensive details of buffer compositions, separation simulations conducted in Simul, and photographic documentation of gels postseparation are provided in the Supporting Information (Table S1, Figures S4 and S5). Here, only the most effective conditions and their corresponding simulations for each counterion are discussed and summarized in Table 1 and Figure 4.

**Table 1:** Conditions of ETP Separation for 0.5% NEEO Agarose Gel

	LE	pH	*c* (mM)	TE	pH of the solution	*c* (mM)	calculated pH in the ETP adjusted zone	DNA recovery
1	HCl Tris	8.3	20	TAPS Tris	8.3	20	8.7	78%
2	HCl Bis-Tris	7.5	20	TAPS Bis-Tris	8	30	8.1	99%
3	HCl BTP	7.7	10	TAPS BTP	7.7	10	8.6	93%
4	HCl His	6.4	20	TAPS His	7.4	20	7.6	80%

**Figure 4:**
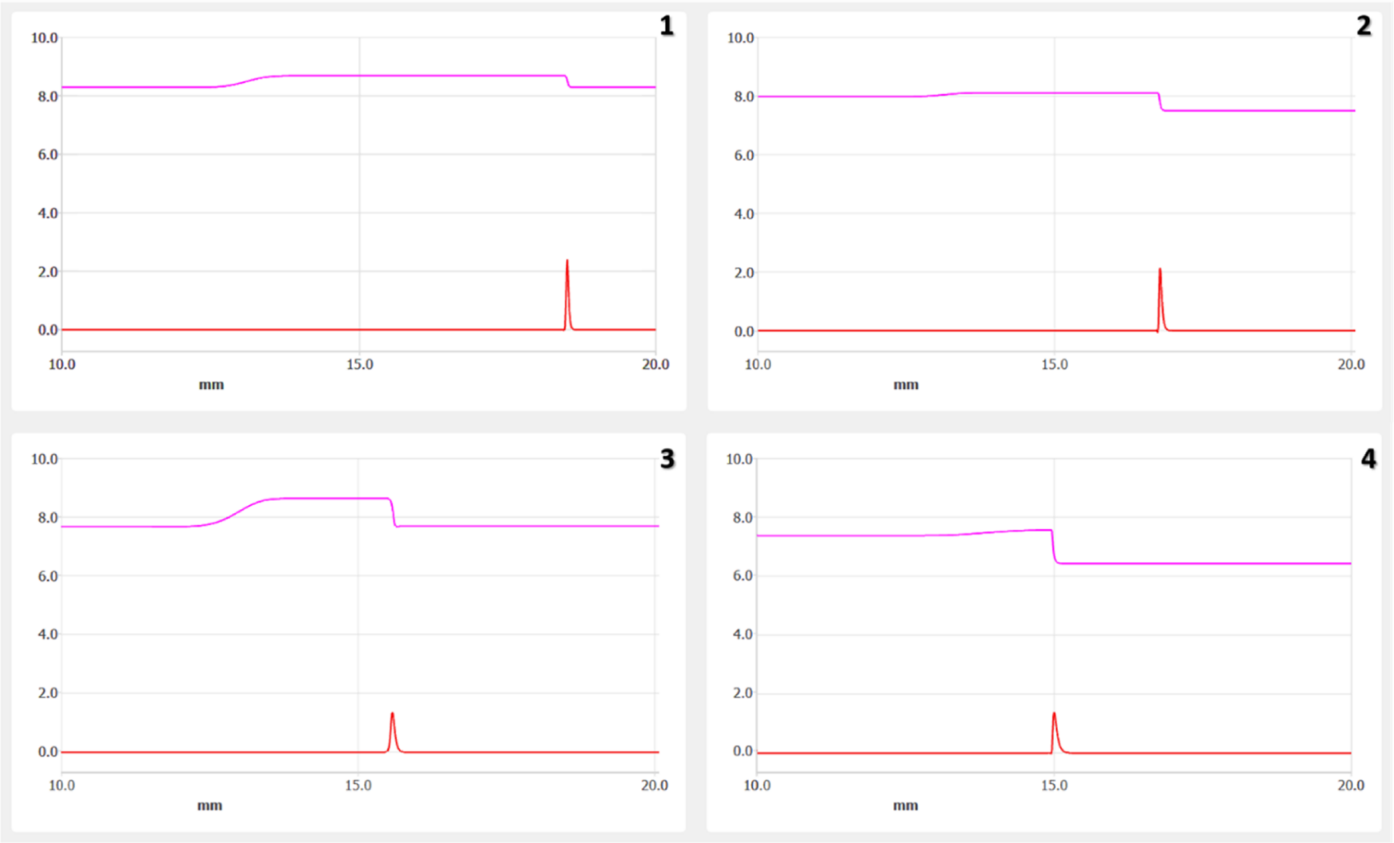
Simulation of the experiments described in Table 1. The red trace indicates the position of the concentrated DNA zone. The purple trace indicates the pH shift. The DNA peaks in the lower traces correspond to the position of the migration boundary between the LE and TE.

For histidine, due to its limited solubility, the maximum achievable pH values were 6.4 for the LE and 7.4 for the TE. It was impossible to find conditions with the histidine cation where gel shrinkage did not occur (Figure S5, photos 24, 25). The Tris counterion, when applied at pH values below 7, lies outside its buffering capacity and exhibits gel shrinkage, likely due to insufficient buffering strength. Conversely, at elevated pH, Tris buffer conditions were optimized, with both the LE and TE set to pH 8.3 at 20 mM concentrations, yielding 78% DNA recovery. A decrease in the TE concentration to 10 mM, under otherwise identical conditions, led to observable gel shrinkage, indicating potential TE ion depletion (see Figure S5, photos 4–6). These findings underscore the necessity of optimizing both pH and buffer concentrations in LE and TE electrolytes. Bis-Tris demonstrated optimal performance near pH 8 (Figure S5, photos 14 and 15), corresponding to the upper limit of its buffering range (5.5–7.5), achieving DNA recovery rates of 99%. The BTP counterion exhibited the highest resistance to gel shrinkage, likely due to its broad buffering range (pH 6–9.5). Multiple favourable conditions were identified, notably, at pH 7.7 with 10 mM LE and TE, and with an increased LE reservoir concentration of 30 mM to expedite separation; DNA recovery reached 93%. A higher pH of 8.3 was also viable, although DNA recovery decreased to between 56% and 75%, depending on experimental parameters (Table S1, Figure S5, photos 20–23). The optimal conditions identified across all tested systems are detailed in Table 1, rows 1–3, with corresponding gel images before (Figure 5, photo 0) and after separation (Figure 5, photos 1–3). For comparison, from the gel shrinking point of view, one nonoptimal electrolyte system with a satisfactory DNA recovery is presented as well, Table 1, row 4, Figure 5, photo 4.

**Figure 5:**
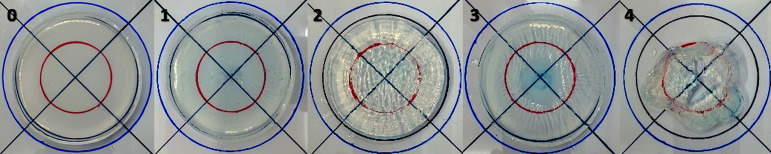
Photographs of 0.5% NEEO agarose gel disks before (0) and after (1–4) ETP separation according to conditions shown in Table 1.

The objective was to establish ETP separation conditions using various counterions that prevent gel shrinkage while maintaining a DNA recovery of at least 80%. Superior results were obtained at neutral to mildly basic pH values, generally situated near the midpoint of each buffer’s effective range. In addition, the potential for TE ion depletion must be considered; therefore, TE concentrations should be maintained at or above those of the LE to ensure reproducibility and efficiency.

### PAA Gels

The PAA gel is commonly used in planar electrophoresis for DNA and protein separations; however, its application has declined due to the carcinogenicity of the acrylamide monomer. In this study, the PAA gel was evaluated as an alternative to agarose gels. Similar to agarose, the lowest workable gel concentration was used to minimize the sieving effects. A relatively low gel concentration of 6% was chosen for the upper concentration range. Separation of Patent Blue and SPADNS dyes was performed under 2 W applied power with a total run time of 109 min. No gel deformation or shrinkage was observed during or after the separation process.

Despite these positive aspects, the PAA gel proved unsuitable for our intended purpose due to the pronounced sieving effect observed with the DNA ladder. Instead of migrating as a single concentrated zone, the DNA ladder has separated into distinct bands corresponding to individual fragment lengths, as illustrated in Figure 6. It is evident from Figure 6 that only the first DNA zone migrated in the epitachophoretic mode, whereas subsequent fragments migrated electrophoretically, with TE acting as the background electrolyte. The sieving effect of the PAA gel caused the DNA separation in two different modes. The fast migrating short fragments, which could freely move through the gel, migrated in the boundary between LE and TE in epitachophoretic mode. Longer DNA fragments, which were sieved by the gel according to their size, could not keep up with the LE/TE boundary and migrated behind this boundary in zone electrophoresis mode. Conversely, this behavior may be advantageous when selective concentration of short DNA fragment sizes is desired. Longer fragments separate into multiple discrete zones over time. The temporal spacing between these zones can be modulated by adjusting the gel pore size, which is inversely related to gel concentration. Further size-based fractionation can be achieved by selecting membranes with appropriate pore sizes in the collection cup, allowing shorter fragments to pass through while retaining larger ones. By combining these strategies, it is possible to selectively collect DNA fragments of a target size while excluding undesired sizes.

**Figure 6:**
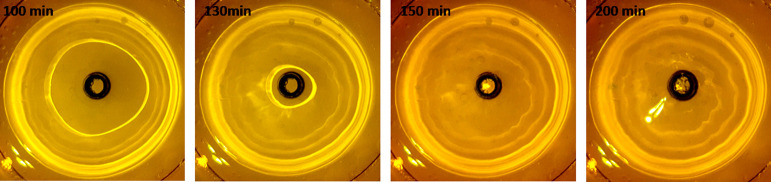
Epitachophoretic/electrophoretic separation of DNA ladder over time. Conditions: 6% PAA gel, LE 20 mM HCl/His pH 6.25, TE 10 mM TAPS/Tris pH 8.3, 1 μg DNA ladder labeled by SYBR Gold, Power = 2 W.

### DNA Recovery

The separation and stabilization of the LE/TE boundary were evaluated not only using dyes but also with a DNA ladder, chosen as a relevant model analyte. DNA recovery was assessed under the following selected conditions: NEEO agarose gel, NEEO agarose gel supplemented with Ba^2+^ ions, IsoGel agarose, and agarose designed for PFGE. In the case of PAA gel, DNA recovery could not be quantified due to the sieving effect. The concentration of DNA recovered from the collection cup, located at the center of the epitachophoretic device, was measured by using a Qubit fluorometer. The DNA concentration time, visualized using SYBR Gold staining, varied depending on the LE/TE stabilization medium composition and the applied power or voltage, ranging from 22 to 109 min. Samples consisting of 0.5 to 1 μL of DNA ladder at 500 μg/mL were diluted into 15–20 mL of TE buffer. After the ETP concentration, the collected volume was 200–400 μL.

Near-complete DNA recoveries were achieved using NEEO and PFGE agarose gel disks, reaching up to 100% recovery (Table 2). All the recoveries presented are the average of at least 3 experiments. The lower recoveries of NEEO agarose under 80% can be caused by the nonoptimal zone formation or worse zone stability caused by the lower buffering capacity of the counterion used at the edge of its buffering capacity. Two exceptions were observed. First one with IsoGel agarose, with the DNA recovery of 16%, where significant DNA retention occurred at the gel disk edges. This theory was confirmed by experiment with SYBR Gold labeled DNA. In this experiment, the majority of the DNA remains at the gel edge and only a tiny zone continues to the center. The second exception was observed with the PAA gel. The pores of the PAA gel were smaller, and a sieving effect occurred. Therefore, the DNA fragments were separated according to their size in zone electrophoretic mode rather than concentrated.

**Table 2:** Stabilization Media for LE/TE Boundary and Its Properties for Epitachophoretic Separation

stabilization media	LE/TE	gel deformation	separation of dyes	power/voltage	*t* (min)	DNA recovery (%)
0–5% NEEO agarose	20 mM HCl.His pH 6–25/10 mM TAPS-Tris pH 8.3	Y	Y	2 W	65	92
1.3% NEEO agarose		N	Y	100 V	60	96
0.5% NEEO agarose +0.5mM Ba(NO_3_)_2_		N	Y	100 V	41	Up to 100
0.3% IsoGel agarose		N	Y	2 W/4 W	93/59	16
0.5% PFGE agarose		N	y	4 W	68	Up to 100
6% PAA		N	Y	2 W	67	-
0.5% NEEO agarose	20 mM HCl–Tris pH 8.3/20 M TAPS-Tris pH 8.3	N	Y	2 W	55	78
	20 mM HCl-Bis-Tris pH 7.5/30 mM TAPS-Bis-Tris pH 8	Y	Y	2 W	50	99
	10 mM HCl-BTP pH 8.3/10 mM TAPS-BTP pH 8.3	N	Y	2 W	31	75
	10 mM HCl-BTP pH 7.7/10 mM TAPS-BTP pH 7.7	N	Y	2 W	35	93
	10 mM HCl-BTP pH 8.3/10 mM TAPS-BTP pH 7.7	N	Y	2 W	30	72

## Conclusions

The stability and physicochemical properties of the electrolyte stabilization media are critical for achieving effective separation, concentration, focusing, and, most importantly, collection of the target analyte in ETP. We tested various gel media for their ability to maintain a stable LE/TE boundary during separation. Initial experiments using dyes helped identify optimal conditions with minimal or no gel shrinkage, which were subsequently applied to achieve a DNA concentration with excellent recovery.

Several types and modifications of agarose gels were evaluated. Despite efforts to select agarose types with a minimal EOF and without ionic surface groups, shrinkage still occurred during separation. The best performance—with negligible shrinkage—was observed using agarose designed for PFGE and IsoGel agarose. However, both gels present drawbacks: high cost and, in the case of IsoGel, retention of larger DNA fragments.

NEEO agarose exhibited a notable shrinkage during separation. Multiple potential causes were investigated, including degradation due to localized heating, EOF from surface ionic groups, and pH or conductivity jump at the LE/TE boundary. EOF was mitigated by the addition of Ba^2+^ ions, which interact with sulfate groups on the agarose chains. While effective at reducing shrinkage, this approach introduces additional components into the system. Adjusting the pH and selecting appropriate counterions for LE and TE also helps to minimize shrinkage. Ideally, the pH of the migrating TE should be close to that of the prepared LE with a well-buffering counterion. Finally, the increased ionic strength of the electrolytes also reduces gel shrinkage, as demonstrated by the addition of physiological saline. From a practical point of view, the best solution for gel shrinkage is the selection of agarose without the EOF. When this is not possible, careful selection of LE and TE will be necessary. Samples with high salt content will decrease or even eliminate the gel shrinking at the expense of an increased run time.

Although the exact mechanism behind the shrinkage of NEEO agarose is not fully resolved, several practical strategies to minimize it have been identified. Another important consideration is the sieving effect of the PAA gel. This property can be advantageous for separating analytes of differing sizes but may hinder recovery if not adequately controlled. When broad analyte size ranges are involved, using stabilization media with large pore sizes, such as agarose, is necessary to minimize the sieving effect.

In summary, successful analyte separation in discontinuous buffer systems such as ITP or ETP requires not only careful optimization of the electrolyte composition but also the appropriate selection and stabilization of the gel media. Using DNA as a model analyte, recovery of close to 100% could be achieved, demonstrating the robustness and effectiveness of the ETP technique.

## Supplementary Material



## Data Availability

All the relevant data are available on 10.57680/asep.0638102.

## Notes

Photographs of NEEO agarose gels after ETP separation, dependence on NaCl addition shown in Figure 3, dependence of NEEO agarose gel shrinkage on the concentration of Ba^2+^ ions, buffering ranges of tested goods’ buffers, experimental conditions of LE and TE composition optimization, corresponding simulation of isotachophoretic separation, and photographs of the NEEO agarose gels after ETP separation (PDF)
