# Supplementary material for: Gel Shrinkage in Discontinuous Electrophoresis: How to Stabilize the Electrolyte Boundary in Epitachophoresis—Part 1—Gel Selection
Source: ACS Omega. 2025 Nov 20;10(48):59513–21. doi: 10.1021/acsomega.5c08736 (PMC12771427; doi:10.1021/acsomega.5c08736)
Supplement: Supplementary file 1 [file ao5c08736_si_001.pdf]

Supporting Information

Gel shrinkage in discontinuous electrophoresis:

How to stabilize the electrolyte boundary in Epitachophoresis – part 1 – gel selection.

Vanda Kocianová[1,2], Ivona Voráčová[1], Doo Soo Chung[3], František Foret[1]

[1] Institute of Analytical Chemistry of the CAS, Veveří 97, 602 00 Brno, Czech Republic

[2] Department of Biochemistry, Faculty of Science, Masaryk University, Kamenice 5, 625 00 Brno, Czech Republic

[3] Department of Chemistry, Seoul National University, Seoul 08826, Republic of Korea

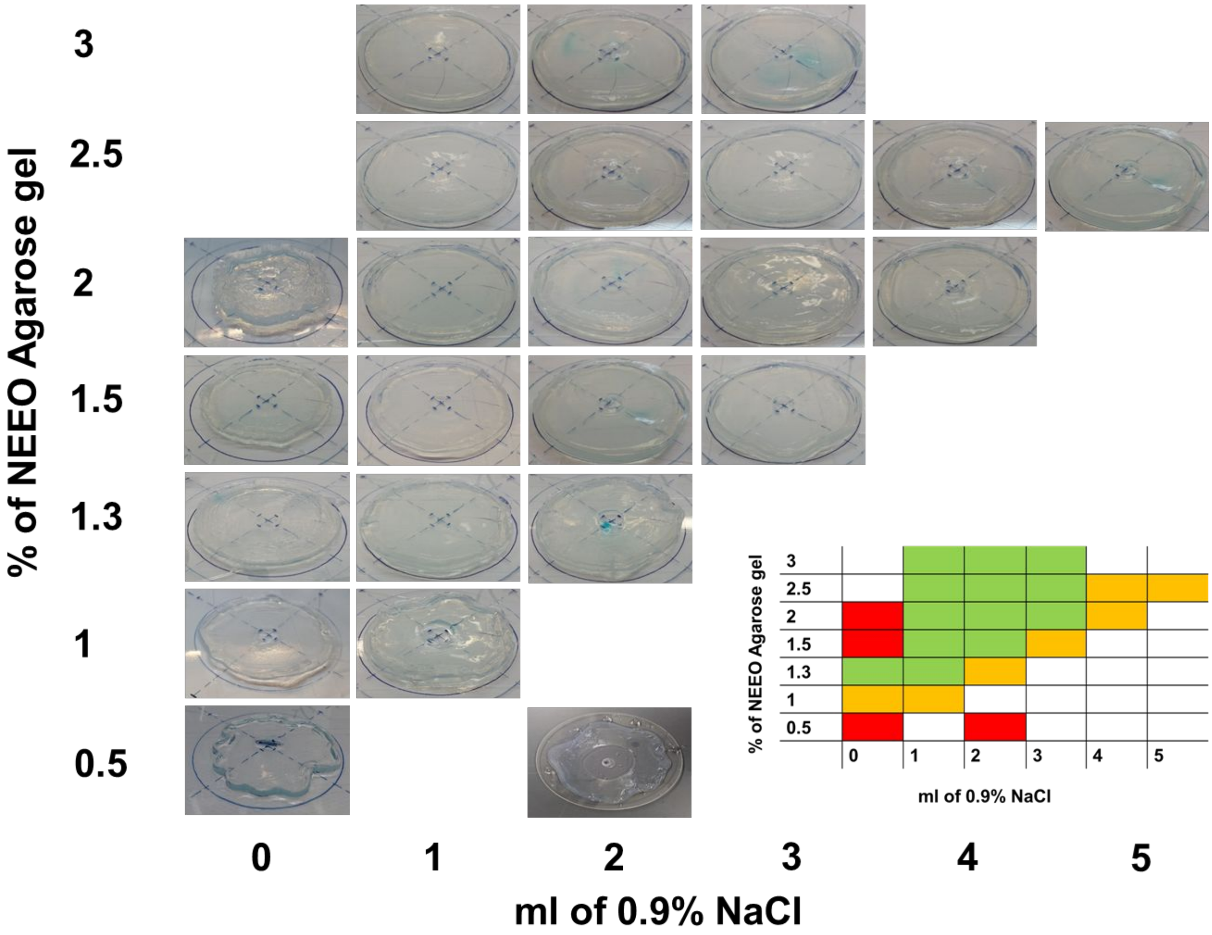

Figure S1. Photographs showing the dependence of NEEO agarose gel shrinkage after ETP separation on the concentration of agarose gel and NaCl addition.

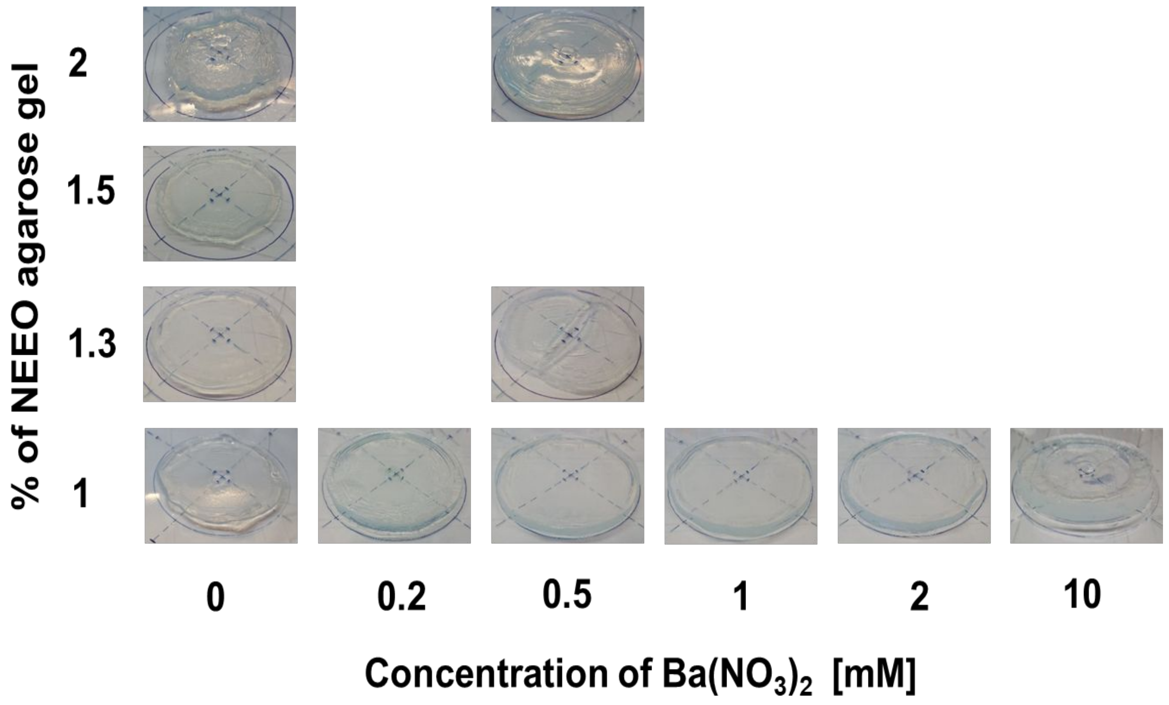

Figure S2. Photographs showing the dependence of NEEO agarose gel shrinkage after ETP separation on the concentration of agarose gel and Ba^{2+} ions

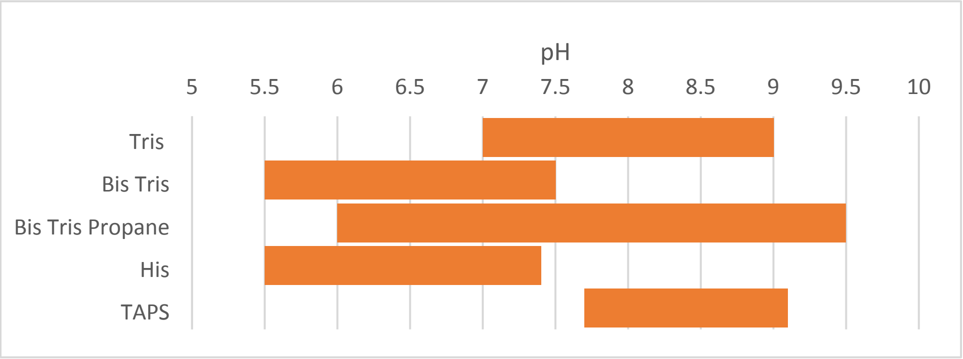

Figure S3. Buffering ranges of the tested Good's buffers

Table S1. Experimental conditions of the optimization of LE and TE composition.

Number of experiment	LE	pH	c_{LE} (mM)	TE	pH	c_{TE} (mM)	R_{DNA} (%)
1	HCl/Tris	6.4	20	TAPS/Tris	8.3	20	-
2	HCl/Tris	7.7	20	TAPS/Tris	7.7	20	-
3	HCl/Tris	7.7	20	TAPS/Tris	7.7	30	-
4	HCl/Tris	8.3	10	TAPS/Tris	8.3	10	-
5	HCl/Tris	8.3	20	TAPS/Tris	8.3	10	-
6	HCl/Tris	8.3	20	TAPS/Tris	8.3	20	78
7	HCl/Bis-Tris	6.7	20	TAPS/Bis-Tris	7.8	20	-
8	HCl/Bis-Tris	7.0	10	TAPS/Bis-Tris	7.2	10	-
9	HCl/Bis-Tris	7.0	20	TAPS/Bis-Tris	7.2	20	-
10	HCl/Bis-Tris	7.0	10	TAPS/Bis-Tris	7.7	10	-
11	HCl/Bis-Tris	7.0	20	TAPS/Bis-Tris	7.7	20	-
12	HCl/Bis-Tris	7.5	10	TAPS/Bis-Tris	8.0	10	-
13	HCl/Bis-Tris	7.5	20	TAPS/Bis-Tris	8.0	20	-
14	HCl/Bis-Tris	7.5	20	TAPS/Bis-Tris	8.0	30	99
15	HCl/Bis-Tris	7.5	30	TAPS/Bis-Tris	8.0	30	-
16	HCl/Bis-Tris propane	6.4	20	TAPS/Bis-Tris propane	8.3	10	-
17	HCl/Bis-Tris propane	7.7	10	TAPS/Bis-Tris propane	7.7	10	93
18	HCl/Bis-Tris propane	7.7	10	TAPS/Bis-Tris propane	8.3	10	-
19	HCl/Bis-Tris propane	8.3	10	TAPS/Bis-Tris propane	7.7	10	72
20	HCl/Bis-Tris propane	8.3	10	TAPS/Bis-Tris propane	8.3	10	56
21	HCl/Bis-Tris propane	8.3	10	TAPS/Bis-Tris propane	8.3	10	75
22	HCl/Bis-Tris propane	8.3	20	TAPS/Bis-Tris propane	8.3	10	-
23	HCl/Bis-Tris propane	8.3	20	TAPS/Bis-Tris propane	8.3	20	-
24	HCl/His	6.4	10	TAPS/His	7.4	10	-
25	HCl/His	6.4	20	TAPS/His	7.4	20	-

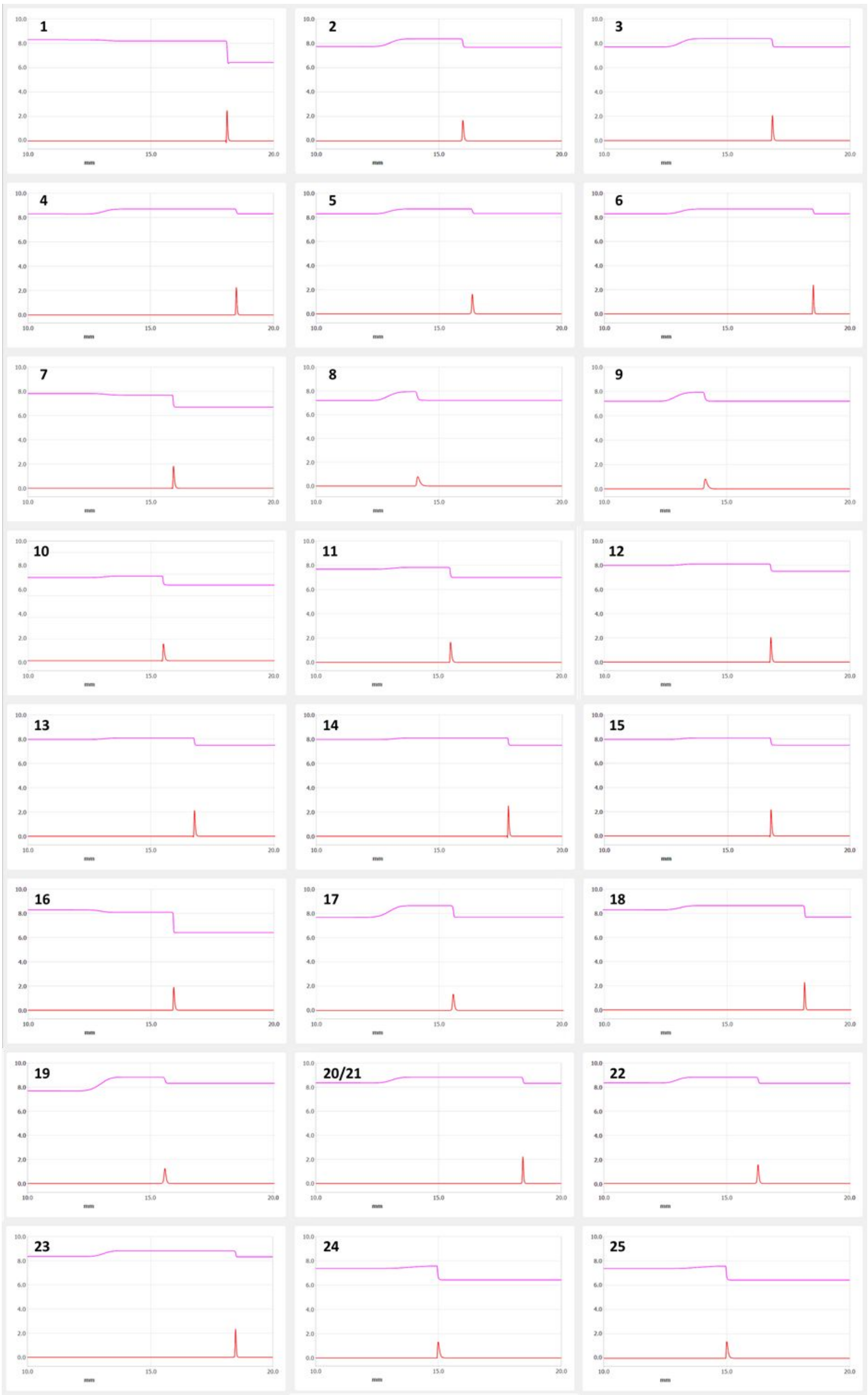

Figure S4. Simulation of the isotachophoretic separation under experimental conditions listed in Table S1; the simulation number corresponds to the experiment number in Table S1.

Vertical axis represents the pH value.

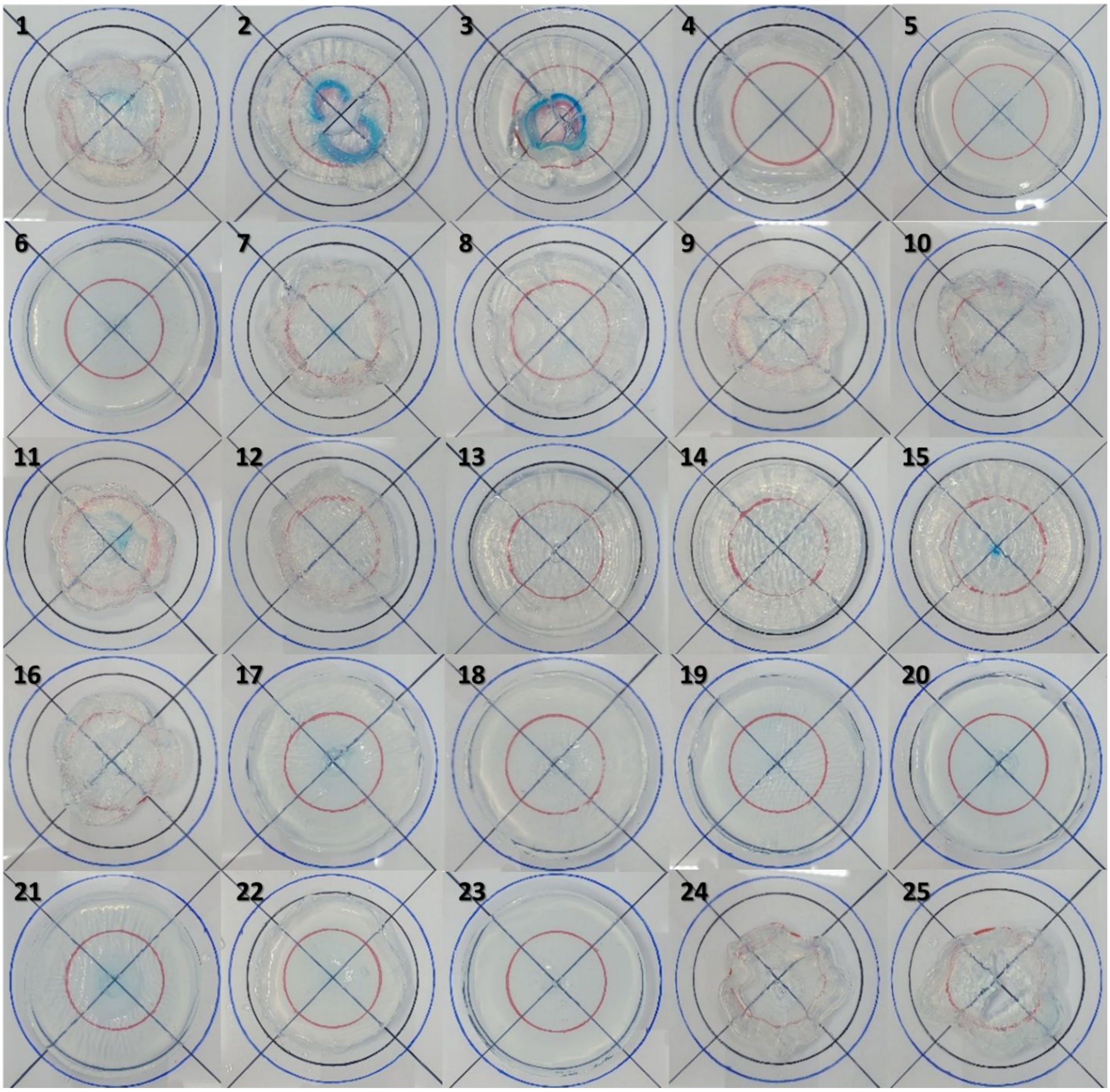

Figure S5. Photographs of 0.5% NEEO agarose gel after ETP separation run according to conditions shown in Table S1, the gel number corresponds to the experiment number listed in Table S1.
